# Larvicidal and anti-termite activities of microbial biosurfactant produced by *Enterobacter cloacae* SJ2 isolated from marine sponge *Clathria* sp.

**DOI:** 10.1038/s41598-023-42475-6

**Published:** 2023-09-13

**Authors:** Sekar Harikrishnan, Shanmugam Sudarshan, Kandasamy Sivasubramani, M. S. Nandini, Jayaraman Narenkumar, Vasudevan Ramachandran, Bader O. Almutairi, Paulraj Arunkumar, Aruliah Rajasekar, Singaram Jayalakshmi

**Affiliations:** 1 Centre of Advanced Study in Marine Biology, Faculty of Marine Sciences, Annamalai University, Parangipettai, Tamil Nadu 608502 India; 2 Department of Aquatic Environment Management, TNJFU- Dr. M.G.R Fisheries College and Research Institute, Thalainayeru, Tamil Nadu 614712 India; 3 Department of Microbiology, Faculty of Science, Annamalai University, Annamalai Nagar, Chidambaram, Tamil Nadu India; 4 Department of Microbiology, Sree Balaji Medical College and Hospital, Chennai, Tamil Nadu India; 5 Department of Environmental & Water Resources Engineering, School of Civil Engineering (SCE), Vellore Institute of Technology, Vellore, Tamil Nadu 632014 India; 6 Department of Medical Sciences, University College of MAIWP International, Taman Batu Muda, 68100 Batu Caves, Kuala Lumpur Malaysia; 7 Department of Conservative Dentistry and Endodontics, Saveetha Dental College and Hospitals, Saveetha Institute of Medical and Technical Sciences, Saveetha University, Chennai, India; 8 Department of Zoology, College of Science, King Saud University, 11451 Riyadh, Saudi Arabia; 9 School of Chemical Engineering, Chonnam National University, Gwangju, South Korea; 10 Environmental Molecular Microbiology Research Laboratory, Department of Biotechnology, Thiruvalluvar University, Serkkadu, Vellore, Tamil Nadu 632115 India

**Keywords:** Environmental monitoring, Environmental microbiology, Bacterial toxins

## Abstract

The widespread use of synthetic pesticides has resulted in a number of issues, including a rise in insecticide-resistant organisms, environmental degradation, and a hazard to human health. As a result, new microbial derived insecticides that are safe for human health and the environment are urgently needed. In this study, rhamnolipid biosurfactants produced from *Enterobacter cloacae* SJ2 was used to evaluate the toxicity towards mosquito larvae (*Culex quinquefasciatus*) and termites (*Odontotermes obesus*). Results showed dose dependent mortality rate was observed between the treatments. The 48 h LC_50_ (median lethal concentration) values of the biosurfactant were determined for termite and mosquito larvae following the non-linear regression curve fit method. Results showed larvicidal activity and anti-termite activity of biosurfactants with 48 h LC_50_ value (95% confidence interval) of 26.49 mg/L (25.40 to 27.57) and 33.43 mg/L (31.09 to 35.68), respectively. According to a histopathological investigation, the biosurfactant treatment caused substantial tissue damage in cellular organelles of larvae and termites. The findings of this study suggest that the microbial biosurfactant produced by *E. cloacae* SJ2 is an excellent and potentially effective agent for controlling *Cx. quinquefasciatus* and *O. obesus*.

## Introduction

Tropical nations are home to a disproportionate number of illnesses transmitted by mosquitoes^1^. The relevance of mosquito-borne diseases is widespread. More than 400,000 people die each year from malaria, and serious illness epidemics from dengue, yellow fever, chikungunya, and zika have been reported in several metropolitan areas^2^. There is an association between vector-borne illnesses and one-sixth of all infections globally, with the most significant occurrence being tied to mosquitoes^3, 4^. *Culex*, *Anopheles*, and *Aedes* are the three mosquito genera most frequently implicated in the transmission of illness^5^. Dengue fever is an infection spread by the vector *Aedes aegypti* mosquito^6^, and its prevalence has been on the rise over the last ten years, making it a significant threat to public health^4, 7, 8^. According to the World Health Organisation (WHO), more than 40% of the world’s population is at risk of developing dengue fever, and 50–100 million new infections occur each year in over 100 countries^9–11^. Dengue has become a significant public health problem since its incidence has increased globally^12–14^. *Anopheles gambiae* Giles, often known as the African malaria mosquito, is the most important vector of human malaria in tropical and subtropical areas^15^. The West Nile Virus, St. Louis encephalitis, Japanese encephalitis, and viral infections of horses and birds are all spread by the *Culex*, often known as common home mosquitoes. Aside from that, they are known to spread bacterial and parasite diseases^16^. There are more than 3000 species of termites around the globe, and they have been there for over 150 million years^17^. Most pest species live in the soil and eat wood and wood products that contain cellulose. A prominent pest, Indian white termite, *Odontotermes obesus* causes significant damage to essential agricultural crops and forest plantation trees^18^. In agricultural regions, termites inflict tremendous economic losses to various crop plants, tree species, and construction materials when infested at different stages. Termites may also create human health issues^19^.

The challenge of medication resistance gained by microbes and insect pests in today's pharmaceutical and agro-based sectors is difficult^20, 21^. As a result, both businesses should search for new cost effective antimicrobials and safe bio-pesticides. Synthetic pesticides are now available and have been proven to be infective and hinder non-target beneficial insects^22^. In recent years, biosurfactant research has expanded recently due to its use in various industrial activities. Biosurfactants are highly beneficial and vital in agriculture, soil remediation, oil recovery, bacterial and insect removal, and food sectors, among other things^23, 24^. Biosurfactants, or microbial surfactants, are biologically surface-active chemicals generated by microorganisms such as bacteria, yeast, and fungus in coastal habitats and oil-contaminated regions^25, 26^. Chemically manufactured surfactants and biosurfactants are the two types that are derived directly from natural settings^27^. Various biosurfactants are obtained from marine habitats^28, 29^. Consequently, scientists are looking for new biosurfactant manufacturing techniques based on naturally available bacteria^30, 31^. The advancement of such research demonstrates the importance of these biological compounds in environmental protection^32^. *Bacillus*, *Pseudomonas*, *Rhodococcus*, *Alcaligenes, Corynebacterium* and those bacterial genera are represented among the well-studied members^23, 33^.

Biosurfactants are extremely varied and have a wide range of uses^34^. These compounds' significant advantage is that some exhibit antibacterial, larvicidal, and insecticidal activities. This implies they may be used in the agricultural, chemical, pharmaceutical, and cosmetic sectors^35–38^. Since they are often biodegradable and ecologically beneficial, biosurfactants have been utilised in integrated pest control programmes to protect crops^39^. Therefore, to obtain fundamental knowledge on larvicidal and anti-termite activities of microbial biosurfactant produced by *Enterobacter cloacae* SJ2. We examined the mortality and histological changes at exposure to different concentrations of the rhamnolipid biosurfactant. In addition, we evaluate the widely used Quantitative Structure–Activity Relationship (QSAR) computer programme, Ecological Structure–Activity Relationship (ECOSAR) to derive the acute toxicity of microalgae, daphnids, and fish.

## Results

**Figure 1:**
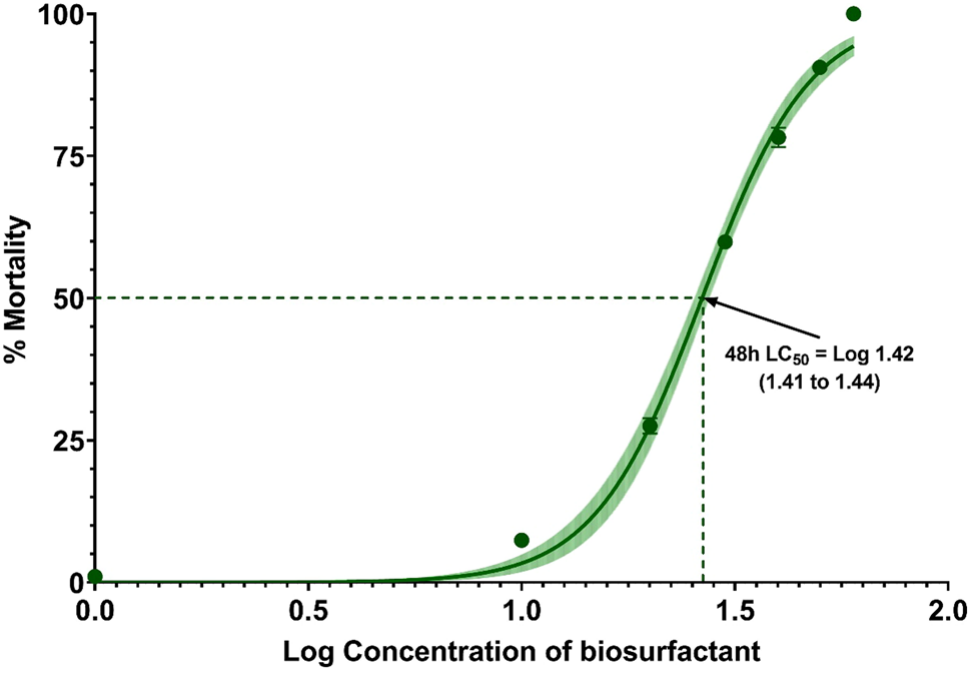
Calculation of LC_50_ values for larvicidal activity. Non-linear regression curve fit (solid line) and 95% confidence intervals (shaded area) on the relative mortality (%).

**Figure 2:**
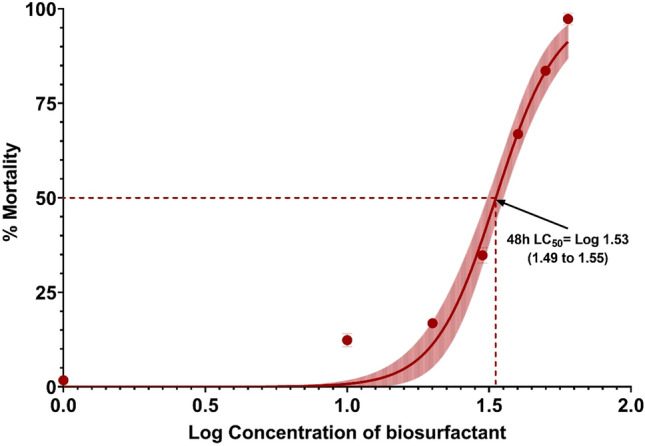
Calculation of LC_50_ values for anti-termite activity. Non-linear regression curve fit (solid line) and 95% confidence intervals (shaded area) on the relative mortality (%).

In the present study, the anti-termite activity (toxicity) of the purified biosurfactant at varying concentrations ranged from 30 to 50 mg/mL with an interval of 5 mg/mL was evaluated against the Indian white termites, *O. obesus* and the 4th instar larvae of *Cx. quinquefasciatus* mosquito larva. The 48 h LC_50_ concentration of the biosurfactant against the *O. obesus* and the *Cx. quinquefasciatus*. Mosquito larva was determined using the non-linear regression curve fit method. The results showed increased termite mortality with the increased concentration of the biosurfactant. Results showed larvicidal activity (Fig. 1) and anti-termite activity (Fig. 2) of biosurfactants with 48 h LC_50_ value (95% CI) of 26.49 mg/L (25.40 to 27.57) and 33.43 mg/L (31.09 to 35.68) respectively (Table 1). In terms of acute toxicity (48 h), biosurfactant belongs to the tested organisms’ “harmful” category. The biosurfactant produced in the present study showed excellent larvicidal activity with a 100% mortality rate within 24–48 h of exposure.

**Table 1:** 48 h LC_50_ values (mg/L) for biosurfactants on anti-termite and larvicidal activity (LC_50_ Median lethal concentration, 95% CI lower–upper confidence interval).

	Anti-termite	Larvicidal activity
LC_50_ mg/L (95% confidence interval)	33.43 (31.09 to 35.68)	26.49 (25.40 to 27.57)
R^2^	0.9731	0.9940
Classification according to United Nations, 2011	Harmful	Harmful

**Figure 3:**
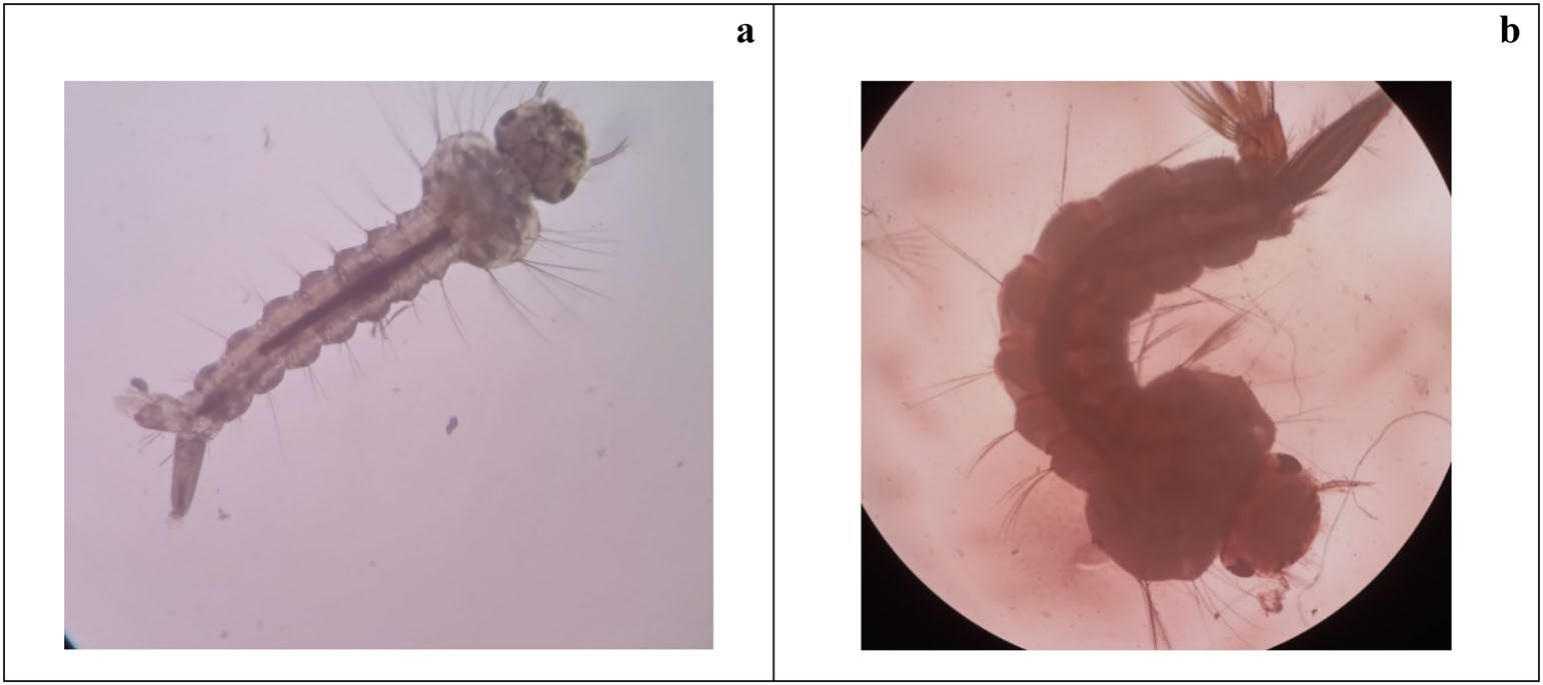
Effect of sub-lethal (LC_50_) dose of biosurfactant on *Culex quinquefasciatus* larval development. Light microscope images at 40 × magnification (**a**) Normal *Cx. quinquefasciatus* (**b**) Abnormal *Cx. quinquefasciatus* larvae.

Under the microscope, morphological changes and abnormalities were observed at the end of the experiment. Control and treated groups at 40 × magnification showed morphologic changes. Most of the larvae treated with biosurfactant revealed growth disruption, as demonstrated in Fig. 3. Figure 3a displays a normal *Cx. quinquefasciatus,* and Fig. 3b shows abnormal *Cx. quinquefasciatus* larvae.

**Figure 4:**
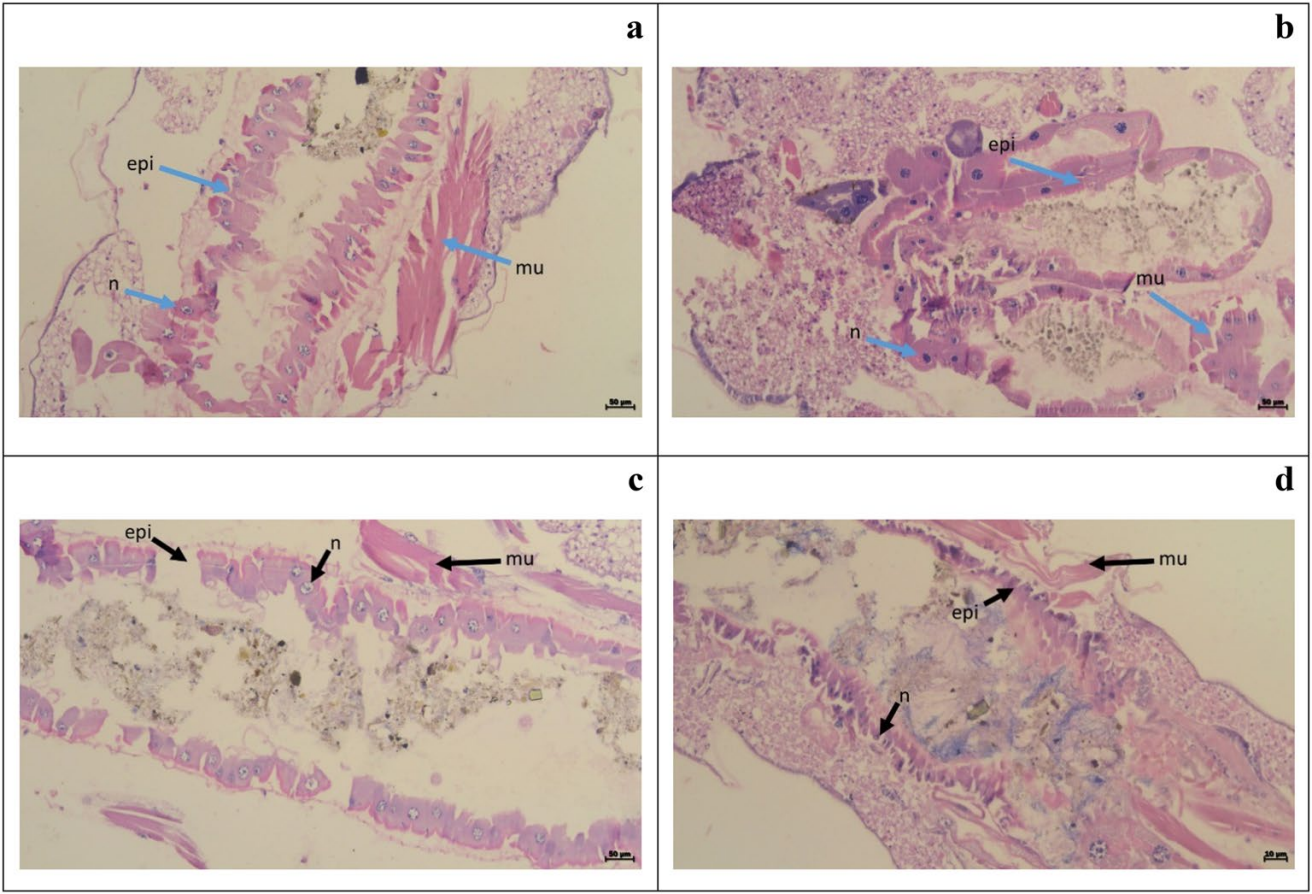
Histopathology of normal untreated 4th instar larvae of *Cx. quinquefasciatus* larva (Control: (**a**,**b**)) and treated with the biosurfactant (Treatment: (**c**,**d**)). Arrow indicates the treated gut epithelium (epi), nucleus (n) and muscles (mu). Bar = 50 µm.

**Figure 5:**
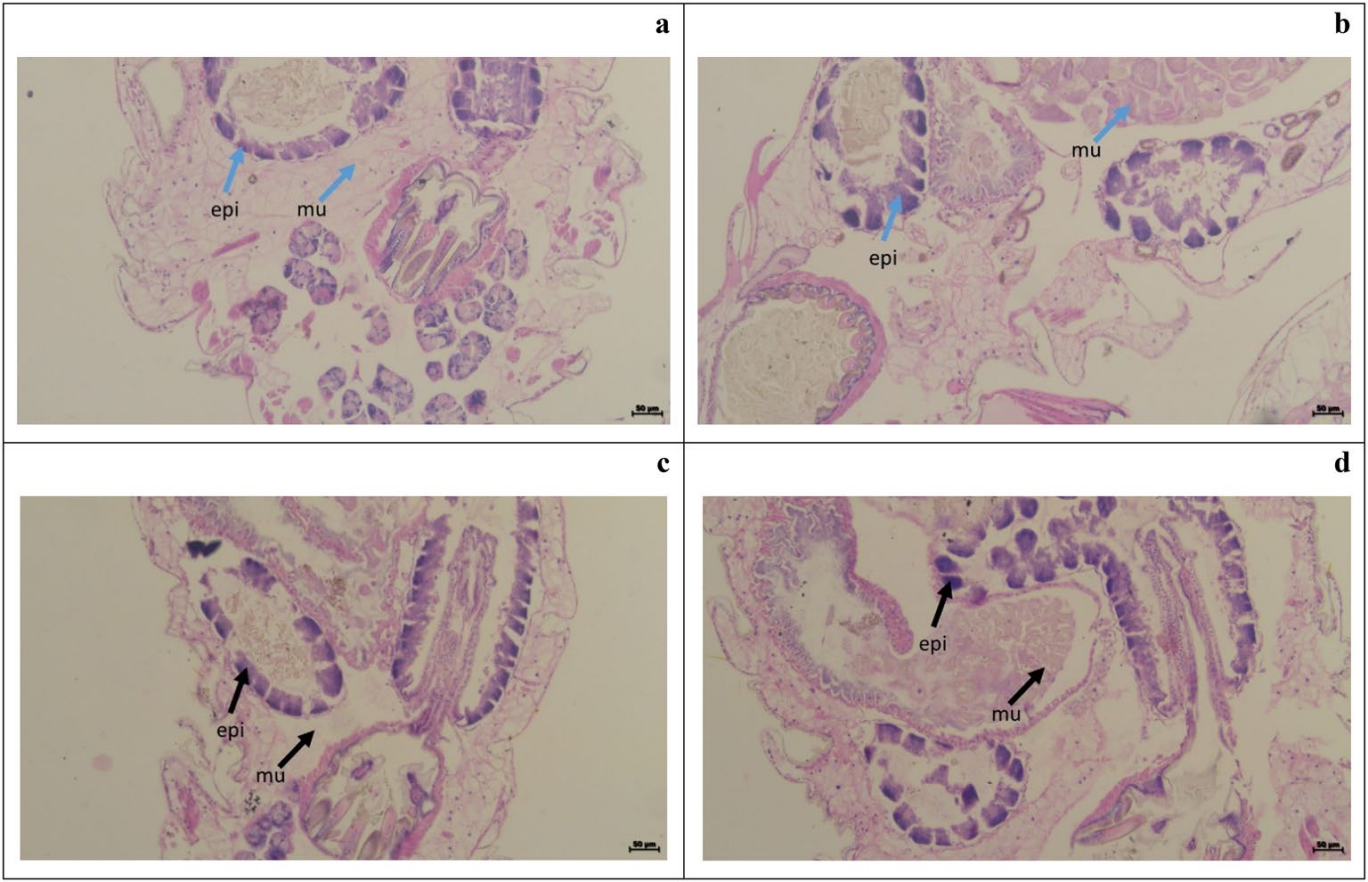
Histopathology of normal untreated *O. obesus* (Control: (**a**,**b**)) and treated with the biosurfactant (Treatment: (**c**,**d**)). Arrow indicates the gut epithelium (epi) and muscles (mu) respectively. Bar = 50 µm.

In the present study, the histological examination of the treated larvae (Fig. 4) and termite (Fig. 5) revealed several abnormalities, including a reduction in the size of the abdominal area as well as damage to the muscles, epithelial layers, and midgut. Histology revealed the mechanism behind the inhibitory activity of biosurfactants used in the present study.

The acute toxicity of rhamnolipid biosurfactant products top primary producer (green algae), primary consumer (daphnia) and secondary consumer (fish) were predicted in this work using ECOSAR. This programme employs sophisticated quantitative structure–activity connection models to assess toxicity based on molecular structure. Using structure activity relationship (SAR) software, the model calculates a substance's acute and long-term toxicity to aquatic species. In particular, Table 2. Summarizes several species’ estimated median lethal concentration (LC_50_) and median effective concentration (EC_50_). Globally Harmonized System of Classification and Labelling of Chemicals was used to classify the estimated toxicity into four levels (Table 3).

**Table 2:** Toxicity evaluation using Ecological Structure Activity Relationship programme (ECOSAR™) for rhamonolipid biosurfactant (*F* fish, *D* daphnid, *A* green algae).

Biosurfactant [CAS number]	Chemical structure and molecular formula	Molecular weight [g/Mol]	ECOSAR™ acute toxicity (mg/L)	Classification according to United Nations^83^
l-rhamnosyl-3-hydroxydecanoyl-3-hydroxydecanoic acid [37134-61-5]		504.7	F (96 h LC_50_): 4.05 mg/L	F: Toxic
			D (46 h LC_50_): 3.13 mg/L	D: Toxic
			A (96 h EC_50_): 8.39 mg/L	A: Toxic

**Table 3:** Classification into defined toxicity classes^83^.

Toxicity range (mg/L)	Class
< 1	Very toxic
1–10	Toxic
10–100	Harmful
> 100	Not classified for acute/long-term hazard

## Discussion

Controlling vector-borne diseases, especially from mosquitoes and strains like *Ae. aegypti*, is a difficult job nowadays^40–46^_._ While some chemically accessible pesticides, such as pyrethroids and organophosphates, are beneficial to some extent, they pose a major hazard to human health, including diabetes, reproductive diseases, neurological dysfunction, cancer, and respiratory illnesses, among other things^4, 8, 47^. Further, eventually, these insects develop resistance against them^13, 43, 48^. Thus, efficient and eco-friendly control measures from biological means will be a more welcome approach to mosquito control^49, 50^. Benelli^51^ suggest controlling mosquito vectors at the early instar stage will be more effective in urban areas, while they do not recommend larvicides in rural areas^52^. Tome et al.^53^ also proposed that controlling the mosquito at the immature stage would be a safe and easy strategy because they will be more sensitive to the control treatments^54^.

The biosurfactant production of the potent strain (*E. cloacae* SJ2*)* showed consistent and promising efficiency. In our previous study reported that the biosurfactant production by *E. cloacae* SJ2 was optimized using physicochemical parameters^26^. According to their study, the optimum condition of biosurfactant production for the potential isolate *E. cloacae* was 36 h incubation, 150 rpm agitation, pH 7.5, 37 °C, 1 ppt salinity, 2% glucose as carbon source and 1% yeast extract as nitrogen source, produced 2.61 g/L of biosurfactant. Furthermore, the biosurfactant properties were characterised using TLC, FTIR, and MALDI-TOF-MS. Which is confirmed that rhamnolipid biosurfactant. Glycolipid biosurfactants are among the most intensively studied classes among the other types biosurfactants^55^. They are comprised of carbohydrate and lipid moieties, mainly chains of fatty acids. Among the glycolipids, rhamno and sophoro lipids are the main representatives^56^. Rhamnolipid comprises two rhamnose sugar moieties linked to mono or di β-hydroxy decanoic acid^57^. Applications of rhamnolipids in medical and pharma industries are well known^58^, besides their use as a pesticide recently^59^.

The interaction of the biosurfactants with the respiratory syphon's hydrophobic area increases the larvae's exposure to the aqueous medium by allowing water to pass through their spiracular cavity. The biosurfactants’ presence also impacts tracheas, which have a range near the surface, making it easier for the larvae to climb to the surface for breathing. As a result, the water surface tension decreases. Because they cannot stick to the water’s surface, larvae drop to the bottom of the tank, which interferes with hydrostatic pressure, leading to excessive energy consumption and death by drowning^38, 60^. Ghribi^61^ reported similar results, that biosurfactant produced by *Bacillus subtilis* demonstrated larvicidal activity against *Ephestia kuehniella*. Similarly, the larvicidal activity of *Cx. quinquefasciatus* larvae by cyclic lipopeptide was also evaluated by Das and Mukherjee^23^.

The results of the present study on the larvicidal activity of a rhamnolipid biosurfactant against *Cx. quinquefasciatus* mosquito are supported by the findings of previous reports^62, 63^. For example, the application of surfactin-based biosurfactant produced from various bacteria belonging to *Bacillus* spp. and *Pseudomonas* spp. on mosquitocidal activity has been reported by several early reports^64–66^, mosquito larvicidal activity of a lipopeptide biosurfactant from *B. subtilis*^23^. Deepali et al*.*^63^ found a rhamnolipid biosurfactant at 10 mg/L isolated from *Stenotrophomonas maltophilia* with potent larvicidal activity. Silva et al.^67^ reported the larvicidal activity of a rhamnolipid biosurfactant at the concentration of 1 g/L against *Ae. aegypti*. Kanakdande et al.^68^ reported that lipopeptide biosurfactant produced by *Bacillus subtilis* results in total mortality of *Culex* larvae and termites with the lipophilic fractions of Eucalyptus. Similarly, Masendra et al.^69^ reported n-hexane and EtOAc lipophilic fractions of *Eucalyptus pellita extract* exhibit 61.7% mortality on worker termites (*Cryptotermes cynocephalus* Light.).

Parthipan et al.^70^ reported the insecticidal application of lipopeptide biosurfactants produced by *B. subtilis* A1 and *Pseudomonas stutzeri* NA3 against the malaria-causing Plasmodium parasite vector *Anopheles stephensi*. They observed longer pupal duration, shorter oviposition period, infertility, and reduced longevity of both larvae and pupae when treated with different concentrations of biosurfactant. The observed LC_50_ values for the biosurfactant from *B. subtilis* A1 were 3.58, 4.92, 5.37, 7.10 and 7.99 mg/L, respectively, for different states of larva (i.e.) larva I, II, III, IV and pupae stage. In contrast, it was 2.61, 3.68, 4.48, 5.55 and 6.99 mg/L for larva stages I-IV and pupae stage, respectively, for the biosurfactant of *Pseudomonas stutzeri* NA3. The delayed phenology of surviving larvae and pupa is thought to be the outcome of significant physiological metabolical abnormalities caused by the insecticidal treatments^71^.

The biosurfactant produced by *Wickerhamomyces anomalus* CCMA 0358 strain showed 100% larvicidal activity against *Ae. aegypti* in 24 h intervals^38^ than that reported by Silva et al*.* wherein the biosurfactant was produced from *Pseudomonas aeruginosa* using sunflower oil as a carbon source showed 100% elimination of the larvae in 48 h^67^. Abinaya et al.^72^ and Pradhan et al*.*^73^ have also confirmed the larvicidal action or insecticide of surfactants produced by several isolates of the genus *Bacillus*. A likely 100% mortality of mosquito larvae exposed to plant limnoids was reported in a previous research published by Senthil-Nathan et al.^74^.

The assessment of the sublethal effects of insecticides on insect biology is critical for integrated pest management programmes because sublethal doses/concentrations do not cause insect death but may reduce insect populations of future generations through interference with biological traits^10^. Siqueira et al.^75^ Observed a complete larvicidal activity (100% mortality) of a rhamnolipid biosurfactant (with 300 mg/ml) when tested at different concentrations ranging from 50 to 300 mg/mL against pyrethroid-resistant and susceptible *Ae. aegypti* strains at the larval stage. They analyzed the mortality time and the effects of sub lethal concentrations through the survival and swimming activity of the larvae. In addition, they observed reduced swimming rate at the sub-lethal concentrations of biosurfactants, such as with 50 mg/ml and 100 mg/mL, after 24–48 h of exposure. Toxicants exerting promising effects in sub-lethal effects are considered to be more effective in exerting multitude levels of damage to the exposed pests^76^.

Histological observation of our results revealed the biosurfactant produced by *E. cloacae* SJ2 significantly altered the tissues of mosquito larvae (*Cx. quinquefasciatus*) and termites (*O. obesus*). Similar kinds of abnormalities was caused by *Ocimum kilimandscharicum* oil formulation on *An. gambiae*s.s and *An. Arabiensis* was reported by Ochola^77^. Kamaraj et al.^78^ also describes identical morphological anomalies in *An. stephensi* larvae exposed to gold nanoparticles. Vasantha-Srinivasan et al.^79^ also reports that *Piper beetle* essential oil severely damaged the hut-lumen and epithelial layers of *Ae. aegypti*. Ragavendran et al. reported that, mosquito larvae treated with the 500 mg/mL mycelia extract of the indigenous fungus *Penicillium* sp. showed severe histological damage in *Ae. aegypti* and *Cx. quinquefasciatus*^80^. Earlier, Abinaya et al. examined the 4th instar larvae of *An. stephensi* and *Ae. aegypti* treated with *Bacillus licheniformis* exopolysaccharide discovered many histological alterations, including the stomach caeca, muscles shrinking, and injured and disordered nerve cord ganglia^72^. According to Ragavendran et al. after being treated with *P. daleae* mycelium extract, the midgut cells of the examined mosquitoes (4th instar larvae) exhibited swelling in the gut lumen, decreased intercellular contents, and nucleus degeneration^81^. Identical histological alterations were also observed in mosquito vector larvae treated with *Acanthospermum hispidum* leaf extracts indicating insecticidal potentials of treated compounds^50^.

Internationally, the application of ECOSAR software has been acknowledged^82^. According to present study, ECOSAR acute toxicity of biosurfactants to microalgae (*C. vulgaris),* fish and daphnid (*D. magna)* belong to the category of “toxic” as defined by the United Nations^83^. The ECOSAR eco-toxicity model uses SARs and QSARs to forecast substances’ acute and long-term toxicity, and it is often used to predict the toxicity of organic pollutants^82, 84^.

## Materials and methods

### Chemicals

Paraformaldehyde, Sodium Phosphate Buffer (pH 7.4), and all other chemicals used in this study were purchased from HiMedia Laboratories, India.

### Biosurfactant production and process optimization

Biosurfactant production was carried out in a 500 ml conical flask containing 200 ml of sterile Bushnell Haas medium supplemented with 1% crude oil as the sole carbon source. The pre-culture of *Enterobacter cloacae* SJ2 was inoculated (1.4 × 10^4^ CFU/mL) and incubated at 37 °C in an orbital shaker at 200 rpm for seven days. After the incubation period, the biosurfactant was extracted by centrifugation of culture medium at 4 °C for 20 min at 3400×*g*, and the resultant supernatant was utilized for screening purposes. Biosurfactant optimization and characterization procedures were adopted from our earlier studies^26^.

### Insect collection and maintenance

The larvae of *Culex quinquefasciatus* was were obtained from Centre of Advanced Study (CAS) in Marine Biology, Parangipettai, Tamil Nadu (India). The larvae were kept in plastic containers having deionized water and were maintained at 27 ± 2 °C under and a photoperiod of 12:12 (light: dark). The mosquito larvae were fed with 10% glucose solution.

### Bioassays of larvicidal action

#### Larvicidal activity

*Culex quinquefasciatus* larvae were found in the septic tanks that were left open and unprotected. A standard classification manual was used to identify and raise the larvae in the laboratory^85^.The WHO recommendations were followed for conducting the larvicidal test^86^. *Cx. quinquefasciatus* larvae of the fourth instar were collected in three sets of 25 numbers in 50 mL of water in a closed test tube with an air gap of two-thirds of its capacity. Biosurfactant (0–50 mg/mL) was applied to each tube individually and kept at 25 °C. Only distilled water (50 mL) was used in the control tube. A dead larva was considered to be one that showed no signs of swimming throughout the incubation phase (12–48 h)^87^. The percentage of larval mortality was calculated using Eq. (1)^88^.

1 $$\mathrm{\% \,Mortality}=\frac{\mathrm{\%\, survival \,in \,control }-\mathrm{\%\, survival \,in \,control }}{\mathrm{\%\,survival\, in \,control }} \times 100.$$

#### Anti-termite activity

The family Odontotermitidae includes the Indian white termite, *Odontotermes obesus*, found in the rotting logs at Agriculture Campus (Annamalai University, India). This biosurfactant (0–50 mg/mL) was tested to see whether it was hazardous using the usual procedure. After drying in a laminar airflow for 30 min, each strip of Whatman paper was coated with concentrations of 30, 40, or 50 mg/mL of biosurfactant. Pre-coated and uncoated paper strips were tested and compared in the centre of the Petri dish. There were around thirty active *O. obesus* termites in each Petri dish. The control and test termites received wet paper as a food source. All the Petri dishes were kept at room temperature throughout the incubation period. After 12, 24, 36, and 48 h, the termites had died off^89, 90^. Termite mortality percentages at various biosurfactant concentrations were then estimated using Eq. (2).

2 $$\mathrm{\%\, Mortality}=\frac{\mathrm{Number\, of \,termite \,dead}}{\mathrm{number \,of \,larval\, introduced}} \times 100.$$

### Larval histology studies

#### Fixing the material

The samples kept on ice and packed in microtubes containing 100 mL of 0.1 M sodium phosphate buffer (pH 7.4) were sent to the Histology Laboratory of the Central Aquaculture Pathology Laboratory (CAPL) at Rajiv Gandhi Centre for Aquaculture (RGCA), Sirkali, Mayiladuthurai District, Tamil Nadu, India for further analysis. The sample was immediately fixed in 4% paraformaldehyde for 48 h at 37 °C.

#### Dehydration and packaging

After the fixing phase, the material was washed thrice with 0.1 M sodium phosphate buffer, pH 7.4, dehydrated in an ascending series of ethyl alcohol, and soaked in LEICA resin for seven days. After that, the substance was placed in plastic moulds filled with resin and polymerizer and then in an oven set at 37 °C until the blocks containing the substance were fully polymerized.

#### Block sectioning/hematoxylin and eosin (HE) staining

Following polymerization, the blocks were sectioned in a LEICA RM2235 Microtome (Rankin Biomedical Corporation 10,399 Enterprise Dr. Davisburg, MI 48,350, United States) with a thickness of 3 mm. The portions were gathered on glass slides, six parts per slide. The slides were dried at room temperature before being stained with hematoxylin for 7 min and washed for 4 min in running water. In addition, the eosin solution was applied to the skin for 5 min and washed for 5 min with running water.

#### Toxicity assessment

Aquatic organisms from various tropical levels were used for predicting acute toxicity: 96 h fish LC_50_, 48 h *D.* magna LC_50_ and 96 h green algae EC_50_. The toxicity assessment of rhamnolipid biosurfactant for fish and green algae was carried out using ECOSAR software for Windows, Version 2.2, developed by US EPA. (Available online at https://www.epa.gov/tsca-screening-tools/ecological-structure-activity-relationships-ecosar-predictive-model).

### Statistical analysis

All larvicidal and anti-termite activity tests were performed in triplicate. The larval and termite mortality data were subjected to non-linear regression (log dose–response variable parameter) to calculate the median lethal concentration (LC_50_) with 95% confidence limits to produce concentration–response curves using Prism® (Version 8.0, GraphPad Software Inc., USA)^84, 91^.

## Conclusion

The current research reveals the potential of the microbial biosurfactant generated by *E. cloacae* SJ2 as a possible mosquito larvicidal and anti-termite agent, and this work will lead to a better understanding of the mechanism behind the larvicidal and anti-termite action. Histological study of larvae treated with biosurfactant showed damage in the digestive tract, midgut, cortex, and hyperplasia of gut epithelial cells. The results revealed toxicological evaluation of rhamnolipid biosurfactant produced by *E. cloacae* SJ2 using anti-termite and larvicidal activity revealed that this isolate was a potential biological insecticide controlling mosquito (*Cx. quinquefasciatus*), and termite (*O. obesus)* vector-borne diseases. It is necessary to understand the basic environmental toxicity of biosurfactants and their potential impacts on the environment. This study provides a scientific basis for the environmental risk assessment of biosurfactants.

## Data Availability

All data generated or analysed during this study are included in this published article.
